# Awareness and perceived risk of cardiovascular disease among individuals living with rheumatoid arthritis is low: results of a systematic literature review

**DOI:** 10.1186/s13075-019-1817-y

**Published:** 2019-01-22

**Authors:** Olivia R. Ghosh-Swaby, Bindee Kuriya

**Affiliations:** 1 Schulich School of Medicine and Dentistry, Western University, London, ON Canada; 2 Sinai Health System, Division of Rheumatology, University of Toronto, Toronto, ON Canada; 3 Department of Medicine, Division of Rheumatology, University of Toronto, Mount Sinai Hospital, The Joseph and Wolf Lebovic Building, 60 Murray Street, Room 2-008, Toronto, ON M5T 3L9 Canada

**Keywords:** Rheumatoid arthritis, Cardiovascular disease, Knowledge, Awareness, Risk factors

## Abstract

**Background:**

Individuals with rheumatoid arthritis (RA) are at risk of developing cardiovascular disease (CVD), but patient perceptions of CVD are not routinely assessed. We performed a systematic literature review to evaluate awareness of the association between RA and CVD, and perceived risk of CVD among individuals with RA.

**Methods:**

Three electronic databases (MEDLINE, EMBASE, and PubMed) were searched for English language articles between the years of 1990–2018. Search terms pertained to RA, CVD, knowledge, awareness, or perceptions of CVD risk. Abstracts were screened for inclusion/exclusion by two independent reviewers.

**Results:**

A total of 33 abstracts were screened and 6 underwent full review. The overall sample size was 478 subjects and included patients with established RA who were predominantly female with a mean age range of 53 to 64 years. RA disease characteristics relevant to CVD were not uniformly reported, including the use of DMARDs, corticosteroids, or NSAIDs. A high proportion of subjects (range 73 to 97%) were unaware of an increased risk of developing CVD in relation to their RA, and this frequently occurred in those with a greater number of traditional CVD risk factors. Misperceptions about CVD were common, and the majority of subjects misestimated their actual CVD risk.

**Conclusion:**

Individuals with RA at highest risk for CVD report low awareness and perceived risk of this comorbidity. This represents a knowledge gap in need of intervention but must be tailored to patients’ needs. An understanding of the system- and individual-level barriers preventing CVD awareness is needed. Only then will approaches to improve CVD screening and management in RA be successful.

**Electronic supplementary material:**

The online version of this article (10.1186/s13075-019-1817-y) contains supplementary material, which is available to authorized users.

## Background

Individuals with rheumatoid arthritis (RA) are at substantially elevated risk for cardiovascular disease (CVD). The risk of coronary artery disease, myocardial infarction, and stroke increases by as much as 82%, 68%, and 41%, respectively [1]. CVD often presents atypically in RA patients and occurs at younger ages so detection of clinical events may be missed or delayed [2, 3]. Development of CVD appears to be mediated by the interplay of traditional CVD risk factors (e.g., dyslipidemia or hypertension), as well as systemic inflammation. When combined, this accelerates endothelial dysfunction and promotes atherosclerosis [1, 2, 4]. Despite improved treat-to-target strategies with early disease-modifying anti-rheumatic drug (DMARD) therapy, premature mortality due to CVD persists [5]. Therefore, CVD is an important health concern for patients, families, and practitioners who care for those living with RA.

Rheumatologists have become keenly aware of the association between CVD and RA, in part due to published evidence-based recommendations for screening and management of CVD [6]. Barber et al. developed CVD quality indicators (QI) for use in rheumatology outpatient settings [7]. However, adherence to these QI was found to be low in clinical practice, especially for communication about the link between CVD and RA to primary care physicians and to patients themselves [8]. Poor translation of this risk results in missed opportunities for education and highlights a gap in CVD preventive care.

In order to empower patients to become active participants in CVD prevention strategies, an understanding of their awareness regarding CVD is required first. Little is known about RA patients’ knowledge about the association between RA and CVD, or how their perceived and actual CVD risk may differ. Therefore, our objective was to perform a systematic literature review evaluating knowledge, awareness, or perceived risk of CVD among individuals living with RA.

## Methods

### Search strategy

A systematic literature review was conducted and reported in accordance to the Preferred Reporting Items for Systematic Reviews and Meta-Analyses (PRISMA) guidelines [9]. A bibliographic search was conducted using three databases up to June 20, 2018: MEDLINE, Embase Classic + Embase, and PubMed. The search strategy included relevant indexing terms and keywords for adults living with RA and their knowledge, awareness, or self-perceived risk of CVD. The specific search terms used are available (Additional file 1).

### Study selection and study quality

Two independent reviewers followed the search strategy and screened through title, abstract, and article for selection (O.G., B.K.). Hand searching of bibliographic lists of relevant articles was also conducted. Articles were included if patient knowledge and perception of CVD was described and evaluated. Pre-defined inclusion criteria were as follows: (1) adult population with RA; (2) observational study involving a survey, questionnaire, interview, or focus group; (3) measured and reported patient knowledge, awareness, or perception of CVD risk; and (4) subject matter pertained to CVD. Any discrepancies between reviewers were resolved through discussion until consensus was reached. Studies were not excluded on the basis of quality alone due to the small sample size. Publication language was restricted to English.

### Data extraction and analysis

The main outcome of interest was awareness of the association between RA and CVD. The secondary outcome was self-perceived risk of CVD in RA. When available, self-perceived risk was compared to calculated risk using a validated risk score. The following additional data were extracted if available: author, year of publication, study design, sample size, patient demographics (sex, age, education, marital status, body mass index), RA disease characteristics (disease duration, seropositivity), RA therapies, CVD treatments, accurate identification of traditional CVD risk factors (hypertension, hyperlipidemia, diabetes, smoking, family history of CVD), and preferences to learn more about CVD in relation to RA. Patient characteristics were described as a mean or percent value. Quantitative analyses could not be done due to the limited number of studies and the heterogeneity of data. Therefore, results are descriptively summarized.

## Results

The search strategy identified 33 references, which were first examined on the basis of titles and abstracts. After exclusion, a total of six studies were used in this review (Additional file 1: Figure S1).

Publication dates ranged between the years 2009 and 2017 (Table 1). The studied populations were geographically diverse, with cohorts from Korea, Denmark, the Netherlands, and the USA (Table 1). Characteristics of the included subjects are found in Table 2. The combined sample size was 478 subjects, predominantly women (range 57 to 91%) with mean age range of 53 to 64 years. Completion of post-secondary education varied widely but was only reported in four studies [10–13]. Subjects had established RA with disease duration ranging from 7 up to 19 years. Other RA variables known to influence CVD such as seropositivity and extra-articular disease were not uniformly reported and only one study described a composite measure of RA disease activity [14]. No studies provided systemic markers of inflammation (e.g., C-reactive protein measurement) [14]. Boo et al. reported the proportion of patients receiving RA therapies including DMARDs, biologics, and glucocorticoids [11]. Non-steroidal anti-inflammatory drugs were recorded in three studies with wide variation (range 18–93%) [11–13].

**Table 1:** Characteristics of included studies in the systematic review

Author, year	*n*	Assessment format	Location	Study objective
Bartels, 2016 [10]	15	Interview	USA	Examined CVD preventive care processes from RA patient and provider perspectives to identify targets for future interventions
Boo, 2017 [12]	200	Questionnaire	Korea	Evaluated patient knowledge of CVD and comparison of perceived risk and actual CVD risk
Boo, 2016 [11]	208	Questionnaire	Korea	Determined if patient perceived risk of CVD was associated with actual risk of CVD
Frølund, 2015 [15]	14	Interview	Denmark	Evaluated effectiveness of a nurse-led screening for CVD in RA
van Breukelen-van der Stoep, 2015 [14]	111	Questionnaire	Netherlands	Evaluated self-reported adherence to CVD prevention strategies
John, 2009 [13]	130	Questionnaire	USA	Validated two parallel versions of the Heart Disease Fact Questionnaire-Rheumatoid Arthritis (HDAQ-RA) for use in clinical practice

*Boo et al. 2016 and 2017 analyzed the same patient population

**Table 2:** Baseline patient characteristics (% or mean) for included studies in the systematic review

Characteristic	Bartels, 2016 [10]	Boo^c^, 2016, 2017 [11, 12]	Frølund, 2015 [15]	van Breukelen, 2015 [14]	John, 2009 [13]
	*n* = 15	*n* = 208	*n* = 14	*n* = 111	*n* = 130
Demographics					
Female (%)	67	91	57	78	79
Age, years (mean)	56	53	62	54	64
Married (%)	60	–	57	75	62
Post-secondary education (%)	80	30	–	–	2
BMI, kg/m^2^ (mean)	–	23	–	27	–
Disease characteristics					
Disease activity (DAS28, mean)	–	–	–	2.4	–
RA disease duration, years (mean)	19	7	14	–	16
Positive rheumatoid factor (%)	–	49	–	58	–
Positive anti-CCP antibody (%)	–	32	–	57	–
Extra-articular disease present (%)	–	25	–	–	–
DMARD therapy (%)	–	95	–	–	–
Biologic DMARD therapy (%)	–	5	–	–	–
Glucocorticoid therapy (%)	–	65	–	–	–
NSAID therapy (%)	–	93	–	–	18
High-risk calculated CVD score^a^ (%)	–	14	40	54	–
CVD risk factors					
Diabetes (%)	13	7	–	–	–
Cigarette smoking, ever (%)	50	5	–	14	57
Hypertension (%)	40	40	–	14	–
Hypercholesterolemia (%)	67	–	–	–	–
Physical inactivity (%)^b^	–	76	–	–	–
Prior CVD event (%)	27	4	–	–	15
Parental history of CVD (%)	–	18	–	–	–
Lipid-lowering therapy (%)	–	17	–	–	–
Antihypertensive therapy (%)	–	26	–	–	–
Insulin/anti-diabetic therapy (%)	–	5	–	–	–

*Anti-CCP* anti-cyclic citrullinated peptide antibody, *BMI* body mass index, *CVD* cardiovascular disease, *DAS28* Disease Activity Score 28 Joints, *DMARD* disease-modifying anti-rheumatic drug, *NSAID* non-steroidal anti-inflammatory drug

^a^High-risk CVD score defined as calculated Systematic COronary Risk Evaluation (SCORE) value of ≥ 20, Framingham Risk Score ≥ 10

^b^Physical inactivity = less than three times/week

^c^Boo et al. 2016 and 2017 analyzed the same patient population

The prevalence of traditional CVD risk factors was heterogeneous and was most commonly documented for cigarette smoking and hypertension [10–12, 14, 15]. Boo et al. described a family history of CVD events, and three studies described a self-report of personal CVD events, with prevalence ranging from 4 to 27% [10–13]. The frequency of self-reported CVD treatment for these risk factors was available in two studies that examined the same population [11, 12].

Four studies assessed patient awareness of the association between RA and CVD [10, 13–15]. A high proportion of subjects (ranging from 73 to 97%) were unaware of an increased risk of developing CVD in relation to their RA (Table 3). Gaps in knowledge were seen regarding the role of exercise, corticosteroids, and non-steroidal anti-inflammatory medications (e.g., diclofenac or ibuprofen) on CVD development [12, 13]. Many patients were unaware of the effects of RA or RA therapies on lipid profiles [13]. Furthermore, when knowledge was assessed using the Heart Disease Fact Questionnaire adapted for rheumatoid arthritis, older populations (age > 60) less commonly identified RA as a risk factor for CVD development [13]. Two of these studies reported goal attainment for CVD risk factors, based on the EULAR recommendations for CVD prevention. Adherence to medications and lifestyle recommendations were self-reported. High-risk patients with diabetes, smokers, or those who were obese less frequently reached target blood pressure levels (not met in 45%) and 69% of high-risk patients had elevated low-density lipoprotein cholesterol levels. High-risk patients were also less likely to adhere to prevention strategies such as dietary advice or physical activity recommendations compared to adherence with medications to control CVD risk factors [12, 14].

**Table 3:** Summary of patient self-reported awareness of the increased risk of developing CVD associated with rheumatoid arthritis

Study	Evaluation of awareness	Aware, *n* (%)	Unaware, *n* (%)
Bartels, 2016 [10]	Q: Are you aware of an elevated CVD risk in RA? A: yes or no	4 (27)	11 (73)
Frølund, 2014 [15]	Q: Did you know that there was an increased risk of developing heart disease among patients with rheumatoid arthritis? A: yes or no	4 (29)	10 (71)
van Breukelen-van der Stoep, 2015 [14]	Q: How high do you think your risk of getting a myocardial infarction is? A: high, not more than average, low, or almost zero	3 (3)	57 (97)
John, 2009 [13]	Q: Patients with rheumatoid arthritis are more likely to develop heart disease? A: yes or no	40 (31)	90 (69)

*Q* question posed in the study to evaluate awareness, *A* patient response options

Awareness was reported among patients who had received some form of educational training or via self-directed reading about CVD [10, 13]. Awareness was also noted for those who were actively taking CVD medications [10, 13]. Additionally, these risk-aware patients were more likely to initiate a discussion about CVD risk factor prevention and treatment with their primary care physician (PCP) or rheumatologist [10, 13]. In contrast, patients who were unware of their CVD risk felt that infrequent visits with their PCP attributed to their limited CVD knowledge and that controlling their RA overshadowed efforts to increase awareness of RA-associated comorbidity [10, 15].

Boo et al. reported the agreement between self-perceived CVD risk and actual 10-year CVD risk, calculated by validated risk scores [11, 12]. In their first study, Boo et al. demonstrated that 13.9% of patients were identified as being at high risk for CVD, 39.9% were at moderate risk, and 46.2% were at low risk according to the Systematic Coronary Risk Evaluation (SCORE) [11]. The majority (96.6%) of those at high risk according to SCORE cut-offs actually underestimated their perceived CVD risk [11]. However, the use of antihypertensive or lipid-lowering medications and having a family history of CVD increased the likelihood in the small number of subjects who accurately perceived themselves as being at high risk for CVD development [11]. Interestingly, the presence of traditional CVD risk factors (diabetes, smoking, physical inactivity, obesity) did not affect perceived risk [11]. In the second cross-sectional evaluation of the same population, Boo et al. compared patient evaluated CVD risk to the Framingham Risk Score [12]. Among the 200 subjects surveyed, 39.5% of subjects correctly perceived their CVD risk. A high proportion (60.5%) misjudged their risk, 19.5% underestimating their CVD risk, and 41% overestimating it. Hypertension, diabetes, obesity, and smoking were highly prevalent among those who underestimated their risk [12]. Older patients, males, and those who had been diagnosed with RA at an older age also underestimated their CVD risk compared to their counterparts [12].

Two studies evaluated attitudes toward CVD prevention and management [10, 15]. At the time of diagnosis, patients reported a preference to focus on controlling their RA, rather than be concerned with CVD [15]. Additionally, acuity and complexity of RA often shifted attention away from discussions of CVD risk during encounters with physicians [10]. In instances when an individual had active RA, management of symptoms was the primary focus rather than CVD preventative care [10]. However, the small sample of patients enrolled in the nurse-led CVD screening program by Frølund et al. were generally positive about the screening consultation and felt it brought a sense of relief, motivation, and control over their health [15].

## Discussion

To our knowledge, this is the first systematic review to assess the degree of awareness or perceived risk of CVD among individuals living with RA. Our key finding was a lack of awareness about the association between RA and CVD, and a misperception of the risk of CVD attributable to RA. Of concern, this mismatch between perceived and actual risk occurred most frequently in the group of patients who were at highest risk for CVD development.

The reasons for these gaps in knowledge may be postulated. An earlier study suggested patients’ lack of knowledge about RA-related risk for CVD stemmed directly from low awareness and non-adherence to the CVD prevention guidelines by healthcare providers [16]. In contrast, Bartels al. reported that while nearly half of patients and PCPs were unaware of the RA-CVD link, 100% of rheumatologists were aware of this risk [10]. This implies that published RA-CVD recommendations are resonating with practicing rheumatologists and argues against physician knowledge as a major barrier to knowledge translation to their patients [6, 10]. In this same study, Bartels et al. conceptualized a CVD preventive framework in RA. In it, they highlight that barriers for adoption of CVD preventive care are not only influenced by providers (e.g., role, expertise, assumption of other providers) but also by system-, visit-, and patient-related conditions [10]. As examples, medical record keeping, workflow, time constraints, visit frequency, and acuity of medical problems are all competing demands that must be balanced when considering CVD-preventive approaches in rheumatology settings [10]. Current models of health care delivery are also typically single disease focused [17]. However, the literature does not support that low awareness is due to unwillingness on the part of patients to learn more about CVD. In fact, a common theme that emerged was the desire to first control symptoms of RA before tackling other health issues [10, 15]. In a survey of our own clinical practice, we found that 87% of respondents were interested in learning more about RA and CVD, with a focus on modifiable risk factors, such as exercise and weight management [18]. Respondents were particularly interested in the role of RA medications and heart disease. Despite this, respondents identified barriers to learning more about this topic, with a primary concern being able to cope with pain due to their RA as well as time constraints [18].

According to the Health Belief Model, awareness and perception of risk are important prerequisites for positive behavioral change [12, 19]. In line with this, self-aware patients independently acted on reducing CVD risk factors and were more likely to initiate conversations with their physician and self-advocate for risk factor treatment [12, 15]. In a study of hypertensive patients, those who were aware that increased blood pressure reduces life expectancy were more likely to comply with check-ups and medication adherence [20]. Another study demonstrated a positive correlation between knowledge of CVD and compliance to lifestyle changes (e.g., diet, exercise stress management) and adherence to drug therapy [21]. Impressive results were seen in a study that implemented a community-based, cardiovascular health awareness program targeted at older adults [22]. Investigators successfully showed reduced rates of myocardial infarction and congestive heart failure hospitalizations, as well as greater antihypertensive treatment in the communities randomized to the intervention versus those who did not receive the educational intervention (EI) [22]. Therefore, incremental gains in knowledge may have big advantages, including reduced CVD morbidity and mortality, which would have significant public health and health policy implications [17]. Whether these substantive changes could also occur in RA settings will require further investigation.

In an era where novel patient education tools are increasingly incorporated into clinical practice, acknowledging the learning preferences of patient groups will assist in the design and implementation of RA-CVD care. This is evident in the study of a nurse-led CVD screening consultation program for RA patients [15]. Participants found the program to be a motivator for lifestyle change but felt the duration and content of the consultation had to be adapted to their needs and their illness [15]. Several studies in this review proposed or included possible strategies to promote awareness of CVD risk in RA. Bartels et al. discussed the implementation of a “spring rheumatology campaign” to encourage patients to improve physical activity and engage in CVD education. This could be analogous to how the autumn season cues influenza vaccination and could employ and align with system interventions that are already in place in many rheumatology clinics (e.g., nursing support, educational resources) [10]. They also suggest the development of multidisciplinary healthcare delivery systems, combining evidence-based management of RA paired with primary prevention and early CVD detection [10]. Boo et al. suggested tailored behavioral interventions and educational programs, specifically for RA, that should be available at the time of RA diagnosis [12]. In line with this, Jolly et al. recently tested the utility of a web-based EI to improve CVD risk awareness among RA patients [23]. The EI was developed by content experts and consisted of a 28-min video, which included pertinent issues in RA such as disease control, medications, and exercise and included patient testimonials to provide motivation to the target audience [23]. The short EI resulted in a significant increase in CVD awareness (measured by the HDFQ-RA), and the largest improvements were noted for RA-specific items compared to general heart disease knowledge items [23]. Based on these preliminary data, web-based EI may be a cost-effective strategy to increase CVD knowledge and behavior change in RA. However, in practice, delivery and uptake of online educational content may pose a challenge. Uptake may be facilitated through the use of smartphone applications or incorporation of links into patient-accessed electronic health records. Many rheumatology clinics currently collect patient-reported outcomes via computers or tablets in waiting rooms, and this may represent another opportunity for patients to view and engage in educational content. However, implementation of any module must consider the needs of learners, including language preferences, ease of use, layout, and time commitment [24]. The content must also be adaptable to the changing landscape of evidence-based medicine.

Our review has some limitations worth mentioning. We limited the review to English language studies and the overall pooled sample size was low. Our results focused primarily on concepts and perceptions, which could not be quantified. Therefore, our results could not be combined and are individually summarized. The classification of perceived risk and awareness was heterogeneous, and no standard for testing or assessing knowledge or perceptions was used across all studies. Three studies incorporated a validated knowledge questionnaire, the Heart Disease Fact Questionnaire-Rheumatoid Arthritis (HDFQ-RA), and tested the accuracy of RA-specific questions pertaining to CVD [11–13]. The questionnaire discussed inflammation, exercise, eating, and traditional CVD risk factors [13]. Assessment tools that discuss knowledge in depth may better categorize awareness and perceived risk as well as highlight specific knowledge gaps. Additionally, studies did not uniformly report important characteristics such as disease activity, medication use that may impact CVD (e.g., NSAIDs), or a personal or familial history of CVD events, which may act as an anchor for familiarity with CVD and thus effect knowledge and awareness. The strengths of our study include the collection of data after 2009 in an era where heightened attention to CVD and RA became more common and thus has relevance to current clinical practice. Moreover, the geographic diversity of the study populations included in this review enhances the generalizability of our findings.

## Conclusions

In conclusion, a systematic review of the literature to date suggests that individuals living with RA have low awareness of the association between chronic, systemic inflammation due to RA and the development of CVD. We encourage additional studies and pragmatic trials of RA-specific educational interventions to be conducted. These will help determine if knowledge gains are sustainable and have substantive impact on long-term CVD outcomes in RA. However, based on our findings, these interventions must be flexible in format, be designed with the input of content experts, including patients themselves, and above all, be tailored to meet the needs of patients. A deeper understanding of the system-, physician-, and patient-level barriers preventing optimal awareness of this comorbidity is also needed. Only then will interventions to improve CVD screening and management in RA be truly successful.

## Additional file

Additional file 1: Appendix. Search strategy used for systematic review record retrieval. **Figure S1.** Flow diagram of the search strategy and study selection for inclusion in the systematic review. (DOCX 67 kb)
