# Supplementary material for: Awareness and perceived risk of cardiovascular disease among individuals living with rheumatoid arthritis is low: results of a systematic literature review
Source: Arthritis Res Ther. 2019 Jan 22;21:33. doi: 10.1186/s13075-019-1817-y (PMC6341634; doi:10.1186/s13075-019-1817-y)
Supplement: Supplementary file 1 — Appendix. Search strategy used for systematic review record retrieval. Figure S1. Flow diagram of the search strategy and study selection for inclusion in the systematic review. (DOCX 67 kb) [file 13075_2019_1817_MOESM1_ESM.docx]

**Additional file 1.** Search strategy used for systematic review record retrieval.

Database: Ovid MEDLINE(R) <1946 to June 20, 2018>

Search Strategy:

1. exp Cardiovascular Diseases/di [Diagnosis] (388200)
2. exp Cardiovascular Diseases/pc [Prevention & Control] (179372)
3. exp Cardiovascular Diseases/et [Etiology] (433703)
4. exp Arthritis, Rheumatoid/ (105520)
5. exp Patients/ (57141)
6. exp Patient Education as Topic/ (79922)
7. exp Patient Satisfaction/ (79276)
8. exp Adult/ (6568274)
9. exp Aged/ (2821421)
10. exp Middle Aged/ (3952867)
11. exp Research Subjects/ (16816)
12. exp Risk Assessment/ (230789)
13. exp risk/ (1068447)
14. exp Risk Factors/ (727272)
15. exp Quality Assurance, Health Care/ (302261)
16. exp Health Knowledge, Attitudes, Practice/ (96478)
17. exp Health Care Surveys/ (31822)
18. exp Interviews as Topic/ (54415)
19. exp Attitude to Health/ (372047)
20. exp Preventive Health Services/ (544224)
21. perception.mp (37186)
22. awareness.mp (162424)
23. 5 or 6 or 7 or 8 or 9 or 10 or 11 (6678617)
24. 1 or 2 or 3 (858711)
25. 15 or 16 or 19 or 20 or 21 or 22 (1158328)
26. 12 or 13 or 14 or 17 or 18 (1142598)
27. 4 and 23 and 24 and 25 and 26 (31)

**Figure S1.** Flow diagram of the search strategy and study selection for inclusion in the systematic review.

Records after duplicates removed
(n = 33)

## Identification

## Eligibility

## Included

## Screening

Additional records identified through bibliographic review

(n = 2)

Records identified through database searching
(n = 31)

Records screened
(n = 33)

Records excluded (n = 25)

- Not-RA specific (n = 4)
- Letter to editor (n=1)
- Review paper (n = 3)
- Not outcome of interest (n = 17)

Full-text articles excluded, with reasons (n = 2)

- No reported measure of awareness/perception (n=2)

Full-text articles assessed for eligibility
(n = 8)

Studies included in qualitative synthesis
(n = 6)
